# The Role of Airway Epithelial Cells in Response to Mycobacteria Infection

**DOI:** 10.1155/2012/791392

**Published:** 2012-04-18

**Authors:** Yong Li, Yujiong Wang, Xiaoming Liu

**Affiliations:** ^1^Key Laboratory of the Ministry of Education for the Conservation and Utilization of Special Biological Resources of Western China, Ningxia University, Yinchuan, Ningxia 750021, China; ^2^College of Life Science, Ningxia University, Yinchuan, Ningxia 750021, China

## Abstract

Airway epithelial cells (AECs) are part of the frontline defense against infection of pathogens by providing both a physical barrier and immunological function. The role of AECs in the innate and adaptive immune responses, through the production of antimicrobial molecules and proinflammatory factors against a variety of pathogens, has been well established. Tuberculosis (TB), a contagious disease primarily affecting the lungs, is caused by the infection of various strains of mycobacteria. In response to mycobacteria infection, epithelial expression of Toll-like receptors and surfactant proteins plays the most prominent roles in the recognition and binding of the pathogen, as well as the initiation of the immune response. Moreover, the antimicrobial substances, proinflammatory factors secreted by AECs, composed a major part of the innate immune response and mediation of adaptive immunity against the pathogen. Thus, a better understanding of the role and mechanism of AECs in response to mycobacteria will provide insight into the relationship of epithelial cells and lung immunocytes against TB, which may facilitate our understanding of the pathogenesis and immunological mechanism of pulmonary tuberculosis disease.

## 1. Introduction

The lung is an organ lined by numerous distinct types of epithelial cells in different anatomical regions. Pulmonary epithelium was initially thought of as a complicated physical barrier to block potentially harmful inhaled bacilli and substances from tissue invasion. However, increasing evidence has demonstrated that epithelial cells also play critical functions in initiating and expanding airway host defense mechanisms in the lung and providing the initial defense against inhaled microorganisms. Furthermore, these epithelial cells are capable of not only regulating innate immunity but also producing functional molecules that physically interacted with immunocytes to activate adaptive immunity [1, 2]. These epithelial cell-derived antimicrobial molecules and/or peptides were multifunctional agents capable of linking the innate and adaptive immune responses [3]. In response to invasion of pathogens, airway epithelial cells (AECs) secrete various microorganism killing effectors, such as mucins antimicrobial peptides (AMPs) and reactive oxygen species (ROS), into the airway lumen to control the composition of airway surface liquid (ASL). These cells also produce proinflammatory cytokines, growth factors, and chemokines that recruit and activate phagocytes to the site of infection eliminating pathogens by phagocytosis. These effectors play key roles in innate immunity of the airway against microorganism invasion and pathogenesis of pulmonary inflammatory diseases caused by chronic bacterial infection [4].

*Mycobacterium tuberculosis *(Mtb) is an extremely dangerous pathogen that primarily infects the lung and is known for its capability to escape innate immune effector cells (such as macrophages and airway epithelial cells) causing tuberculosis in humans and animals [5]. The AECs are one of the first host cells encountering invaded mycobacterial pathogens, despite increasing evidence, which demonstrates the potential roles of these cells in the tuberculosis (TB) pathogenesis, as well as the innate and adaptive immune responses against the infection [6–9]. The exact mechanism of epithelial mediated host defense and the clinical relevance of the epithelial cell-mediated immune responses remain poorly understood. This paper focuses on current knowledge regarding the roles of AECs in response to mycobacteria infection. An emphasis was placed on the recently recognized functions of epithelial cells in innate and adaptive immunity against mycobacteria, particularly the pathogen recognition and host defense of AECs to Mtb.

## 2. Airway Epithelial Cells and Pathogen Recognition of Mycobacteria

*Mycobacterium* utilizes multiple strategies for establishing infection of the lung, including adhering, invasion, and replication in alveolar macrophages [6, 10, 11]. An important step of infection is the initial contact of a pathogen to host cells including epithelial cells. The innate immune response is the first line of host defense responsible for immediate recognition and control of pathogen invasion. The lung microenvironment contains an intricate milieu of pattern recognition molecules in the innate immune system that contribute to the host primary response to inhaled pathogens such as Mtb. It has been suggested that Mtb affects the result of the bacillus-host interaction with various recognition molecules [12, 13]. The family of Toll-like receptors (TLRs) and surfactant proteins (SPs) of AECs play a key role in the recognition and binding of the pathogen to epithelial cells during Mtb infection. Additionally, other pattern recognition receptors (PRRs) such as NOD2, Dectin-1, c-type lectin receptors (CLPs), mannose receptor, and DC-SIGN are also believed to be involved in the recognition of Mtb [14].

### 2.1. Role of Epithelial TLRs in the Recognition of Mycobacteria

Genetic association studies revealed that TLR variants contributed to the susceptibility of humans to TB [15]. The genes of various TLRs have been demonstrated to be predominantly expressed in immunocyte such as macrophages, dendritic cells (DCs), B lymphocytes, monocytes, and natural killer (NK) cells. The AECs have also been shown to constitutively express TLRs which are one of the most important PRRs for pathogen recognition. These TLRs mediate the host-pathogen interaction through pathogen-associated molecular patterns (PAMPs) and initiate appropriate signaling before immune cells are recruited to the airways. To date, thirteen distinct mammalian TLRs have been discovered, ten of which recognize PAMPs, and have been identified in humans (designated TLR-1 to 10). The expression of TLR-1, TLR-2, TLR-4, TLR-5, TLR-6, and TLR10 was located at the apical cell membrane of AECs where they mainly recognize proteins, lipoproteins, and the polysaccharides of the bacteria. In addition, the expression of TLR-3, TLR-7, TLR-8, and TLR-9 was detected in cytoplasmic compartments where they scavenge different types of nucleic acids from viruses [16–18]. Each TLR recognizes a unique molecular pattern and a variety of ligands to mediate the production of the appropriate cytokines to produce an effective immune response.

In response to the infection of Mtb, airway epithelial activation of NF-*κ*B and other associated signaling molecules are activated *via *TLR-mediated signaling pathways. Previous studies have demonstrated functional TLR-1-6 and TLR-9 expressed in human bronchial epithelial cells [13] and several TLRs involved in the immune response of AECs against Mtb infection (Table 1). Furthermore, human bronchial epithelium was capable of regulating its sensitivity to recognize microbes by management of the TLR's expression levels to control microbial recognition in mucosal compartments [13]. Mice deficient of myeloid differentiation factor 88 (MyD88), a TLR mediator molecule, were approximately twofold more susceptible to lethal doses of Mtb infection relative to wild-type mice even though MyD88-deficient mice vaccinated with *Mycobacterium bovis *Bacille Calmette-Guérin (BCG) were able to confer substantial protection from acute Mtb infection [19]. This study suggests that MyD88-dependent TLR signaling might be dispensable to adaptive immune responses to Mtb, but crucial for the innate immune response to control Mtb infection. Among these, TLRs, TLR-2, TLR-4, TLR-6, and MyD88 played the most prominent roles in the initiation of the immune response against Mtb [20].

TLR-2 is expressed throughout the human airway epithelium, predominantly in the noncolumnar epithelial and alveolar cells [21], which mediated responses to a variety of bacterial components including the lipoproteins of Mtb, Gram-positive and Gram-negative bacteria [22]. In response to Mtb infection, TLR-2 was specifically targeted to a 19 kDa lipoprotein and lipoarabinomannan of this pathogen, and its activity, ligand specificity, and capacity of signal transduction were mainly determined by a heterodimer formed with TLR-2 and other TLR family members, such as TLR-1 and TLR-6 [23]. It has been suggested that the formation of a TLR-2 heterodimer with TLR-1 or TLR-6 has evolved to expand the ligand spectrum to promote the innate immune system for recognition of numerous structures of lipoproteins and lipopeptides presented by different pathogens [24–26]. TLR-2-deficient mice infected with live mycobacterial aerosol displayed reduced bacterial clearance, a defective granulomatous response, and development of chronic pneumonia. The pulmonary immune responses in TLR-2-deficient mice showed increased levels of IFN-*γ*, TNF-*α*, and IL-12p40, as well as the CD4^+^ and CD8^+^ cell fractions. However, these responses were not sufficient enough to protect the deficient mice from Mtb infection within 5 months after infection suggesting that TLR-2 might be a regulator of inflammation, and the exaggerated immune inflammatory response in these mice resulting from the absence of TLR-2 [27]. In AECs, TLR-2-dependent activation has been demonstrated to function as an indirect role in host defense by inducing the proinflammatory cytokine interleukin (IL) 8 (IL-8) and the antimicrobial peptide human *β*-defensin-2 (HBD-2). The action of HBD-2 was believed to act through the chemokine receptor C-C chemokine receptor 6 (CCR6) to recruit immature dendritic cells (DCs) and memory T cells to the site of infection [28]. The neutrophil chemoattractant, IL-8, recruits these cells to the site of pathogen exposure and eventually eliminates the infection [29]. These processes may contribute to the resulting adaptive immune response at the site of infection [30].

The function of TLR-4 in both the host's innate and adaptive immunities has been extensively studied, as well as TLR-4 expressed in alveolar and bronchial epithelial cells, and increased in the cells activated by TLR-4 ligands [18, 31, 32]. However, it is difficult to distinctly define the exact role of TLR-4 against Mtb infection. TLR-4 mutant (C3H/HeJ) mice intranasally infected with live Mtb were more susceptible to pathogen and development of pulmonary tuberculosis relative to wild-type (C3H/HeN) mice, suggesting that TLR-4 may play a protective role in host defense against lung infection by Mtb [33]. This result was consistent with the finding that TLR4 is critical in controlling chronic Mtb infection in mice [34]. However, a controversial report found that TLR-4-mutant mice exhibited increased resistance to low doses of Mtb infection relative to their wild-type litter mates in a dose-dependent manner [35]. Furthermore, TLR-2/TLR-4/TLR-6-deficient mice showed their capacity to control chronic *M. bovis *infection, indicating that TLR-2, TLR-4, and/or TLR-6, may be redundant in controlling mycobacteria infection [27]. Synergistic interactions of TLR-2, TLR-6 and TLR-9 in AECs induced high levels of antimicrobial activity against *Pseudomonas aeruginosa *infection in mice [36], suggesting that multiple TLRs in AECs may produce innate immune responses by the activation of EGFR *via *a cell signal cascade [37]. These results demonstrate the complicated nature of TLR signaling and hence warrant further studies to establish its protective role against Mtb infection.

### 2.2. Other Pattern Recognition Receptors

Additional epithelial PRRs have been investigated including Dectin-1, CLPs, NOD2, DC-SIGN, and the mannose receptor. Dectin-1, expressed on the surface of myeloid lineage cells, was initially identified as fungal PRR that bound to *β*-glucans and triggered cytokine production by facilitating interaction with TLR-2 [38]. Recent studies that investigated the innate response to mycobacteria demonstrated Dectin-1 expression in alveolar type 2 (ATII) cells that played a critical role in response to Mtb in these non-phagocytic cells [38–40]. A549 cells, an ATII cell line, infected with Mtb resulted in active induction of Dectin-1 in a TLR-2-dependent manner. Furthermore, the induction of Dectin-1 and Mtb-mediated production of ROS was mutually dependent. Additionally, Mtb-dependent Dectin-1 expression was also dependent upon Src kinases. Selective inhibition of Src signaling dramatically decreased Dectin-1 expression. Moreover, Mtb internalization could be partially blocked by silencing Dectin-1 expression, inhibiting Src kinases, or pretreating Mtb with antioxidants [39]. Dectin-1 also has been demonstrated to play a role in promoting Mtb-induced IL-12p40 production, in which it acts to increase bacterial-host cell interaction and thus enhance the subsequent cytokine response in macrophages and DCs [38]. Further studies revealed that Dectin-1 was required for proinflammatory cytokine release and antimicrobial effects on intracellular mycobacterial growth in A549 cells [41].

### 2.3. Surfactant Protein and Mycobacteria Binding

Pulmonary SP is a multimolecular complex comprised of phospholipids and proteins, which are primarily secreted by airway submucosal cells, Clara cells, and ATII cells into to the extracellular and intra-alveolar space. There are four main surfactant proteins that have overlapping functions, known as SP-A, B, C, and D. Additionally, ATII cells enable synthesis of all four SPs and surfactant lipids that are packaged together in a unique secretory organelle known as the lamellar body [42]. SP physiologically acts to reduce the surface tension of the alveoli by allowing expansion of the lung during inspiration, as well as maintaining alveolar stability by reducing surface tension along the epithelial lining [14, 43]. Human SPs exhibit the capacity to bind Mtb and alter human macrophage-mediated functions *in vitro*. Although, recent *in vivo *studies demonstrated no gross defect in SP-A, SP-D, or SP-A/D deficient mice in the uptake or immune control of Mtb. This suggests that SP-A and SP-D were dispensable for immune control of low doses of Mtb challenge *in vivo *[44]; a number of *in vitro *and *in vivo *studies clearly showed that they were involved in host defense functions against Mtb infection [11, 14, 44, 45].

SP-A and SP-D, members of the collectin family, are capable of interacting with pathogens primarily by mediating surfactant function [46, 47]. Previous studies have revealed the ability of SPs to regulate the initial interaction between Mtb and its intracellular niche, the alveolar macrophage [48]. SP-A has been suggested to play a role in surfactant homeostasis and the host's defense in the lung by its involvement in the early capture and phagocytosis of the pathogenic Mbt by alveolar macrophages [49]. Furthermore, SP-A has been shown in a rat model infected with *M. bovis *BCG to enhance mycobacteria killing by macrophages through a nitric-oxide-(NO-) dependent pathway. In this study, SP-A specifically bound to and enhanced the uptake of BCG organisms by macrophages. The ingestion of SP-A-BCG complexes by rat macrophages led to production of inflammatory mediators and increased mycobacteria killing [50]. SP-A preferred to bind the ligands of mannosylated lipoarabinomannan (ManLAM) and lipomannan on the surface of BCG and *M. smegmatis*, without discrimination between virulent and nonpathogenic strains [49]. The alanine- and proline-rich antigenic (Apa) glycoprotein, expressed with restriction in the Mtb complex strains, was another new potential target for human pulmonary SP-A. This glycoprotein is associated with the Mtb cell wall for a significant amount of time enhancing the attachment of SP-A and possibly accounting for the selective recognition of these strains by SP-A and immune system c-type lectins [51].

SP-D is a lectin that recognizes carbohydrates *via *its c-type carbohydrate recognition domains (CRDs), and it has been shown to primarily bind the terminal mannosyl oligosaccharides of Mtb to agglutinate bacilli by bridging the carbohydrate binding domains [11]. The conformation of SP-D is extremely important for binding to bacilli. The 321-glutamic acid (Glu) position of human SP-D is a critical site in the binding and regulation of Mtb-macrophage interactions mediated by recognition of Mtb mannosylated cell wall components [52]. The binding of SP-D and lipoarabinomannan on the surface of Mtb may result in bacterial agglutination, reduced uptake, and bacilli growth impairment within human macrophages. The SP-D-mediated inhibition of intracellular growth of Mtb in macrophages is independent of aggregation and may be a result of increased phagosome-lysosome fusion rather than the generation of a respiratory burst [53, 54].

## 3. Airway Epithelial Cells and Their Host Defenses

AECs serve as the first line of defense against pathogen invasion by their function of structural defense as a physical barrier and initiation and augmentation of airway host defense mechanisms. AECs secrete numerous antimicrobial substances, enzymes, ROS, NO, and proinflammatory chemokines and cytokines in response to invasion of a bacterial pathogen such as Mtb, which constitutes a major part of host defense against pathogen infections in the lung.

### 3.1. Antimicrobial Substances (Peptides)

AECs are able to constitutively secret and/or induce secretion of antimicrobial substances including lysozyme, lactoferrin, defensins, collectins, pentraxins, secretory leukocyte protease inhibitor (SPLI), hepcidin, and cathelicidin (LL-37). These antimicrobial peptides are essential elements of innate immunity. Among them, LL-37, *β*-defensin 2, and hepcidin have been demonstrated to play critical roles in innate immunity against mycobacteria infections.

LL-37 is the only member of the cathelicidin family identified to be expressed in human AECs and alveolar macrophages, and it is a major antimicrobial peptide in the innate immune system against Mtb [55]. The expression of LL-37 has been seen in the A549 cells infected or stimulated with mycobacteria such as BCG. BCG-mediated upregulation was influenced by NADPH/ROS, MEK1/2, and p38 MAPK signaling pathways, which played a central role in the regulation of LL-37 gene expression [56, 57]. Furthermore, the induction of LL-37 expression was also observed with an increasing expression of TLR-2, TLR-4, and TLR-9 signaling in this epithelial cell type [56].

Human *β*-defensin-2 (HBD-2) is another known inducible antimicrobial peptide associated with the pathogenesis of human TB that has the capacity to control the growth and chemotactic activity of Mtb [58]. Following BCG infection, the expression of HBD-2 in human epithelial cells was increased by means of NF-*κ*B modulation of increased TNF-*α* production. In addition to its direct mycobicidal activity, HBD-2 also possesses immunomodulatory functions of stimulating IL-8 production by AECs, as well as enhancing proliferation and cytokine production of CD4^+^ T cell. These studies provide insight into how epithelial-expressed HBD2 enhances the capacity of host defense to control Mtb infection *in vivo *[59].

Hepcidin (gene name hepcidin antimicrobial peptide (*HAMP*)), a key negative regulator of iron metabolism in the body, was originally identified as having antimicrobial properties against bacterial infections. The liver is the primary hepcidin-producing organ, although other tissues such as the lung also synthesize this antimicrobe [60, 61]. Mouse dust cells infected with Mtb exhibit inducible expression of hepcidin mRNA. Similarly, elevated expression of hepcidin may be induced in human alveolar epithelial A549 cells when stimulated by lipoglycans, particularly Mtb mannose-capped lipoarabinomannan, and phosphatidyl-myo-inositol mannosides. The lysed Mtb subcellular fractions and culture filtrate proteins, as well as live BCG, have been shown to stimulate hepcidin mRNA expression in DCs [60]. These results imply that epithelial hepcidin secretion acts as a host defense mechanism against mycobacteria infection.

### 3.2. Airway Epithelial Cell Produced Proinflammatory Factors

Functional PRRs expressed in epithelial cells at different airway mucosal sites provide the capacity of AECs to sense the presence of pathogen infection allowing epithelial cell secretion of various proinflammatory chemokines and cytokines against the pathogen. Mycobacteria activate several signaling events upon contact with AECs that stimulate production of tumor necrosis factor-*α* (TNF-*α*), interleukins, and granulocyte-macrophage colony-stimulating factor (GM-CSF). These proinflammatory factors function to recruit and activate phagocytic cells to eradicate organisms and infected cells [62–64]. Several proinflammatory factors produced by AECs in response to mycobacteria infection are listed in Table 2.

TNF-*α*, a cytokine involved in systemic inflammation primarily secreted by the activated macrophages and lung AECs, stimulates the acute phase reaction. Mtb was able to penetrate alveolar epithelium by compromising the epithelial barrier properties and eliciting the production of TNF-*α* after infection, which in turn reduced the bioelectric properties of alveolar epithelium. This process may result in establishing Mtb infection and pulmonary TB [9]. *In vitro *studies on A549 cells demonstrated that Mtb-induced ROS production may be essential for increased expression of TNF-*α*, IL-6, and IL-8 in AECs. This was believed to be mediated by the activation of mitogen-activated protein kinases (MAPKs)/extracellular signal-regulated kinase (ERK) 1/2 and p38 MAPK signaling pathways [65]. Production of TNF-*α*, elevating expression of IFN-*γ* and its receptor, as well as the mediator of IFN-*γ* signaling pathway, signal transducer and activator of transcription 1 (STAT1) was seen in Mtb-infected A549 cells [66].

AECs have not previously been considered as an important source of chemokines in pulmonary TB. However, a growing body of evidence has shown that the AECs, particularly the alveolar epithelial cells, are a major source of several chemokine secretions involved in the host response to mycobacteria infection. Furthermore, alveolar epithelial cells may also contribute to the local inflammatory response in human TB by producing chemokines that attract monocytes, lymphocytes, and polymorphonuclear (PMN) cells to the site of infection [67, 68]. IL-8 (CXCL8) is an important activator of the host immune response to Mtb by recruiting inflammatory cells to the site of infection. A signaling pathway comprised of Duox1-TACE-TGF-*α*-EGFR on the surface of AECs was suggested as a defense against BCG infection by production of IL-8 [69]. Intracellular growth was also necessary for mycobacteria to stimulate monocyte chemotactic protein-1 (MCP-1, also known as C-C motif ligand 2 (CCL2)) and IL-8 secretion by alveolar epithelial cells. However, the mycobacterial virulence and rate of intracellular growth did not correlate with chemokine production [67]. Multiple TLRs have been shown to play major roles in IL-8 secretion by activation of EGFR *via *cell signaling cascades in AECs [37, 70, 71]. Production of IL-8 in BCG-infected human epithelial cells could be reduced by IL4, another important cytokine in human TB. However, IL-10 production was unaltered by IL-4 in the AECs [72]. The expression of IL-10 was induced by IL-27 in bronchial epithelial cells regulated by the activation of the phosphatidylinositol 3-OH kinase (PI3K)-Akt signaling pathway [73]. Function of IL-27 was related to the pathogenesis of chronic obstructive pulmonary disease (COPD) and patients with pulmonary TB.

IL-18 is a member of the IL-1 cytokine superfamily and is secreted by macrophages and other cell types including human ATII cells. IL-18 plays an important role in the induction of a cell-mediated immunity by binding to its receptor following infection [74]. Human ATII cells are a major resource of IL-18, and a baseline of IL-18 expression could be detected in normal ATII cells. The ATII cells and ATII-derived IL-18 might play a potential role in the pathomechanism of granulomatous in pulmonary TB. This is supported by the previous findings that ATII cells produce IL-18 in primary lung tissue cultures from patients with pulmonary TB [75]. This study found markedly increased IL-18 mRNA levels and increasing amounts of intracellular IL-18 protein with increasing culture time in ATII cell cultures stimulated with Mtb whole cell lysate. Additionally, the expression and secretion of mature IL-18 protein from human ATII cells was altered by the proinflammatory cytokines TNF-*α*, IL-1*β*, and IFN-*γ* [75].

Other inflammatory factors found to be produced in mycobacteria infected airway epithelial cells include granulocyte colony-stimulating factor (G-CSF), GM-CSF, interferon-inducible protein-10 (IP-10, also known as C-X-C-motif chemokine 10 (CXCL10), secreted by cells in response to IFN-*γ*), IFN-*γ*-inducible T-cell *α*-chemoattractant (I-TAC, also called CXCL11 or IP-9, a small chemokine chemotactic for activated T cells), monokine induced by IFN-*γ* (MIG, also known as CXCL9, a T-cell chemoattractant induced by IFN-*γ*), monocyte chemoattractant protein-1 (MCP-1), and leukotriene B4. The epithelial cells also produced inflammatory factors that play important roles in the recruitment of activated T cells and pathogenesis of pulmonary diseases in which IFN-*γ* is highly expressed, such as pulmonary TB [67, 76–78].

### 3.3. Alveolar Epithelial Cell Necrosis and Mycobacterial Infection

Mycobacteria are able to invade and replicate in both macrophages and epithelial cells in the alveolar spaces of the lung. The alveolar epithelial cells actively contribute to the innate immune response in the lung and play an important role in mycobacterial dissemination during primary infection by undergoing cellular necrosis and releasing mycobacteria. Though the mycobacteria infection induces alveolar epithelial cell apoptosis, necrosis is the primary cause of cell death in infected alveolar cells. The necrosis was not a consequence of mycobacterial growth or the production of inflammatory factors in the host cells, but due to increased permeation of the cell membrane and the infection of live bacilli [79, 80]. The suppressed apoptosis in the infected alveolar cells correlated with an upregulation of apoptosis inhibitors bcl-2 and Rb, and a downregulation of proapoptotic genes, BAD and BAX. This finding was opposite to that in mycobacteria-infected macrophages, suggesting that differential induction of apoptosis between macrophages and alveolar epithelial cells exists representing cell-specific strategies used by Mtb for survival in the host [80].

### 3.4. Epithelial Cells Secreted Matrix Metalloproteases and TB Immunopathology

The mechanism of Mtb lung tissue damage and mycobacterial spread remains poorly understood. Previous studies demonstrate that matrix metalloproteases (MMPs) have a unique ability to degrade fibrillar collagen at neutral pH leading to lung matrix destruction [81–85]. MMP activity was dependent upon a monocyte-epithelial cell network and p38 MAPK phosphorylation to achieve the matrix-degrading phenotype [83]. The Mtb 6 kD early secreted antigenic target (ESAT-6) protein was able to induce MMP-9 secretion from the epithelial cells neighboring infected macrophages [86]. Human primary bronchial epithelial cells cultured with conditioned medium from Mtb-infected monocytes (CoMTb) had upregulated levels of MMP-1. This increasing of MMP-1 was mediated *via *phosphorylation of p38 MAPK that was induced by synergism of CoMTb-driving TNF-*β* and G protein-coupled receptor activation, and the decreasing of tissue inhibitor of metalloproteinase 1 (TIMP-1) secretion. Clinically it has been shown that activated p38 localized to the MMP-1-secreting AECs of TB patients [81]. Furthermore, CoMTb was able to upregulate MMP-9 gene expression and secretion in primary human bronchial epithelial cells, while inhibition of the p38 MAPK activity led to decreased secretion of MMP-9. TNF-*α* was necessary, but not sufficient for MMP-9 upregulation by a monocyte-epithelial cell network. This suggests that undefined soluble factors in CoMTb may synergize with TNF-*α* to increase MMP-9 secretion in the epithelial cells [82]. These results clearly imply that host- and pathogen-derived factors may work in concert to Mtb infection to drive MAPK-dependent MMPs secretion from AECs. Increased MMP secretion may lead to enhanced recruitment of macrophages, which contributed to earlier granuloma maturation and bacterial growth. This notion was supported by the fact that disruption of MMP9 function alleviated granuloma formation and bacterial growth [87]. Hence, interruption of the production of epithelial MMPs may be a therapeutic target for treatment of TB.

### 3.5. Airway Epithelial Cells Produce Nitric Oxide against Mycobacteria Infection

Nitric oxide (NO) and reactive oxygen intermediates are toxic molecules used by the immune system. Production of NO by host cells is an effective host defense mechanism against microbial infection. Bose et al. demonstrated that in a murine TB model NO production played an essential role in Mtb killing by mononuclear phagocytes, particularly during the early phase of the infection. Additionally, it may also play a role in tissue damage during the late phases of the disease [88]. *In vitro *studies using A549 cells showed an induction of NO in response to Mtb infection, which is consistent with the finding that NO has antimicrobial effects against Mtb infection [89]. These results suggest that a correlation exists between NO production and innate immunity of AECs against mycobacteria [90].

## 4. Airway Epithelial Cells: The Connection of Innate and Adaptive Immunity

Airway epithelial cells have been recognized to play important roles in the defense of invaded microbial pathogens, *via *both the innate and adaptive immune responses [2, 4]. It has become increasingly evident that AECs express PRRs that recognize microbial pathogens and activate innate host defense mechanisms in the airway. Furthermore, the activated innate response secondarily induces recruitment and activation of DCs, T cells, and B cells that augment antigen recognition, antibody production, and other adaptive immune components. These response mechanisms include epithelial production of cytokines and chemokines that stimulate communication between the “nonprofessional” (epithelial cells) and “professional” (B cells, T cells, DCs, and macrophages) immune cells [4].

Following infection of mycobacteria, the host immune system must first recognize the invading pathogens, activate the innate response, and initiate the adaptive immune response. Though “professional” immune cells play a central role in the initiation of adaptive immunity, the alveolar epithelial cells are among the first cell types to encounter mycobacterial pathogens, implying that crosstalk between alveolar epithelial cells and “professional” immune cells may be important in initiating a coordinated secretion of chemokines and the subsequent recruitment of leukocytes to the lung. ATII cells are capable of producing various antimicrobial and proinflammatory molecules that contribute to pulmonary immunity. These cells do so not only by secreting chemokines that recruit inflammatory cells to the lung but also serving as antigen-presenting cells [91]. Although ATII cells are unlikely to prime naïve T cells, their ability to present antigens to T cells suggest that they may play a role in the effector phase of the immune response [91]. Interestingly, the migration of monocytes across the alveolar epithelial-endothelial barrier required the production of chemokines and the presence of surface molecules on both the alveolar epithelial and endothelial cells [10]. These studies demonstrate a novel role for ATII cells in the immunological response to pulmonary pathogens such as Mtb [91]. However, the interaction between AECs and immune cells (e.g., the macrophages) in response to Mtb infection is currently largely unknown.

## 5. Concluding Remarks

It is well accepted that airway epithelium plays an important role in the innate immune response by its collection of surface, endosomal, and cytosolic sensors that activate numerous proinflammatory signal pathways. Additionally, resident antimicrobial substances secreted by the epithelial cells offer significant mechanisms to deal with the invaded pathogens. A growing body of evidence supports the essential involvement of AECs in both the innate and adaptive immune responses against mycobacterial infection. These studies have facilitated the understanding of the major components of the innate immune system and their role in the host's defense of AECs, despite the fact that the interaction between AECs and Mtb is far less known in comparison with the interplay of alveolar macrophages and this pathogen. A recent study using *Mycobacterium marinum* in the zebra fish model revealed a molecular mechanism of mycobacteria induce granulomas, a hallmark of tuberculosis. In this mechanism model, the mycobacteria secreted ESAT-6 induced MMP-9 in those epithelial cells neighboring the infected macrophages, which in turn contributed to nascent granuloma maturation and bacterial growth by increasing the recruitment of macrophages to sites of infection [86, 87]. Together with the findings of the ability of epithelial cell-secreted SPs to regulate the initial interaction between Mtb and alveolar macrophage, these studies provide an insight into the interaction/crosstalk between macrophages and AEC in pathogenesis of TB [48, 49, 86, 87].

AECs act to sense infectious danger by functionally expressing PRRs. Meanwhile, they are able to limit potentially harmful inflammatory reactions within the framework of organ-specific immunity by increasing the activation threshold [92]. In the case of mycobacterial infection, interaction between the pathogen and AECs results in the production of antimicrobial substances or peptides, lysozyme, lactoferrin, *β*-defensins, and NO. Furthermore, these cells produce and secrete proinflammatory cytokines and chemokines thus initiating the adaptive response by recruitment of inflammatory cells. The mechanisms used by the airway epithelium to discriminate between pathogenic or nonpathogenic *Mycobacterium*, sense pathogens to immune effectors, interaction/crosstalk between AECs and alveolar macrophages, as well as the clinical relevance of epithelial cell-mediated immune responses, have yet to be fully elucidated.

## Figures and Tables

**Table 1:** Evidenced TLR-related AEC in response to Mtb infection.

TLRs	Typical Ligands	Cellular location	References
TLR1	Microbial lipoproteins	Cell membrane	[93, 94]
TLR2	Microbial lipoproteins; PGN; LTA; zymosan;	Cell membrane	[21, 22, 24, 25, 27, 94, 95]
TLR3	dsRNA	Intracellular components	[95–97]
TLR4	LPS; RSV S protein; retroviral envelope protein	Cell membrane	[34]
TLR5	Flagellin	Cell membrane	[27, 33, 35, 98, 99]
TLR6	LTA; zymosan; diacyl lipopeptides	Cell membrane	[27, 93, 100]
TLR7	ssRNA imidazoquinoline	Intracellular components	[95, 101]
TLR8	ssRNA imidazoquinoline	Intracellular components	[101–103]
TLR9	Demethylated CpG DNA	Intracellular components	[60, 98, 101, 104]

LPS: lipopolysaccharide; ssRNA: single-stranded RNA; dsRNA: double-stranded RNA; LTA: lipoteichoic acid; PGN: peptidoglycan; RSV: respiratory syncytial virus.

**Table 2:** Epithelial cell produced chemokines and cytokines against mycobacteria infections.

Inflammatory factor	Producing cells	Reference
TNF-*α*	Alveolar epithelial cells	[9, 65]
IFN-*γ*	Alveolar epithelial cells	[66]
GM-CSF	Bronchial epithelial cells	[105]
IL-1	Airway epithelial cells	[76]
IL-4	Human bronchial epithelial cells	[72]
IL-6	Alveolar epithelial cells	[65]
IL-8 (CXCL8)	Airway epithelial cells, alveolar epithelial cells	[65, 66, 76]
IL-10	Bronchial epithelial cells	[73]
IL-18	Human alveolar type II cells	[74, 75]
IL-27	Bronchial epithelial cells	[73]
IP-10	Alveolar epithelial cells, bronchial epithelial cells	[67, 76–78]
MIG	Bronchoepithelial cells	[78]
I-TAC	Bronchoepithelial cells	[78]
MCP-1	Alveolar epithelial cells	[67, 76, 77]

IP-10: interferon-inducible protein-10; MIG: monokine induced by IFN-gamma; I-TAC: IFN-inducible T-cell alpha-chemoattractant; MCP-1: monocyte chemoattractant protein-1.
